# The Effects of a Mindfulness Program on Mental Health in Students at an Undergraduate Program for Teacher Education: A Randomized Controlled Trial in Real-Life

**DOI:** 10.3389/fpsyg.2021.722771

**Published:** 2021-12-06

**Authors:** Lise Juul, Eva Brorsen, Katinka Gøtzsche, Birgitte Lund Nielsen, Lone Overby Fjorback

**Affiliations:** ^1^Department of Clinical Medicine, Danish Center for Mindfulness, Aarhus University, Aarhus, Denmark; ^2^VIA University College, Aarhus, Denmark

**Keywords:** pragmatic clinical trial (MeSH), community mental health services (MeSH), stress, psychological (MeSH), mental health (MeSH), mindfulness-based stress reduction (MBSR), mindfulness (MeSH), student teachers

## Abstract

**Background:** In this study, we aimed to investigate the effects of a mindfulness program including Mindfulness-Based Stress Reduction (MBSR) on the mental health of student teachers when offered at their educational institution in a real-life context.

**Methods:** A parallel randomized controlled trial (RCT) was conducted among self-selected student teachers at a Danish undergraduate program for teacher education in the autumns of 2019 and 2020. Participation was not recommended in case of (1) clinical depression or a diagnosis of psychosis or schizophrenia, (2) abuse of alcohol, drugs, and/or medicine. Randomization was performed by a Statistician who was blinded to the identity of the students. Data was collected using self-reported questionnaires. The primary outcome was a change in perceived stress 3 months from baseline. Secondary outcome measures were symptoms of anxiety and depression, well-being, resilience, mindfulness, and thoughts and feelings during rest. The effects were analyzed according to the intention-to-treat principle using mixed-effect linear regression models. Mediating effects of mindfulness skills on the mental health outcomes were explored using structural equation modeling.

**Results:** The study group included 67 student teachers with 34 allocated to the intervention group (median age: 25 years; women: *n* = 24, 71%); and 33 students (median age: 25 years; women: *n* = 25, 76%) allocated to a waiting list control group. At baseline, mean Perceived Stress Scale (PSS) scores were 18.88 (*SD*: 5.75) in the intervention group and 17.91 (*SD*: 6.36) in the waiting list control group. A total of 56 students completed the questionnaire at a 3-month follow-up (28 in both the intervention- and the control group). Statistically significant effects of the intervention were found on perceived stress, symptoms of anxiety and depression, well-being, and on three of seven resting-state dimensions. No effects were found on resilience or mindfulness. Statistically significant mediated effects via resting-state dimensions were found.

**Conclusion** The findings suggested that offering a mindfulness program at an undergraduate program for teacher education could significantly improve the mental health among self-selected students within 3 months. Results of mediation analysis supported the hypothesis that some of the effects might be explained by reduced distracting thoughts.

**Clinical Trial Registration:** [www.ClinicalTrials.gov], identifier [NCT04558099].

## Introduction

Poor mental health in young people is a public health challenge in all societies (Patel et al., 2007). Patel et al. (2018) stress the importance of not stigmatizing mental health problems and suggest that societies provide mental health promotion as population-based strategies. This is in line with the Perth Charter for Promotion of Mental Health and Well-being, an outcome of the seventh World Conference on the topic, which emphasizes that mental health promotion should be incorporated throughout the life span via activities and settings such as educational institutions (Clifford Beers Foundation and Mentally Health WA, 2012).

International studies among university and college students have shown that large proportions of students experience high levels of stress, anxiety, and depression (Macaskill, 2013; Duffy et al., 2019; Ramón-Arbués et al., 2020). A population-based Danish health profile from 2017 showed increased perceived stress measured by the Perceived Stress Scale (PSS), especially among the 16–24-year-olds (Larsen et al., 2018). It also showed that students had a higher mean PSS compared to the mean in the total population (Sørensen et al., 2021).

High levels of student stress are associated with the experience of high demands, performance pressure (Fabricius et al., 2019), lack of support from teachers and co-students, as well as lower motivation leading to greater drop-out rates. In this regard, stress is both a barrier to well-being and social affiliation but also to professional development in some students (Danish Evaluation Institute, 2019). Some professions require good mental and social resources as well as highly developed interpersonal skills in order to respond to diversity and stressful situations (Hwang et al., 2019). For example, working as a teacher can be stressful and unpredictable as it involves a high degree of interaction with pupils, their parents, colleagues, and society. Thus, it becomes relevant to identify ways to enhance these resources and competencies in student teachers to prepare them for their future job.

A recent meta-analysis concluded that Mindfulness-Based Programs (MBPs) were the most effective interventions in improving well-being compared to distinct types of psychological interventions (van Agteren et al., 2021). Another recent review and meta-analysis including 51 randomized controlled trials (RCTs) that were conducted among university students, found that MBPs improve mental health by decreasing distress and symptoms on anxiety and depression and improving well-being in university students (Dawson et al., 2020). The same is evident in studies of groups comparable to student teachers like students training to work in the healthcare sector (Daya and Hearn, 2018; Chen et al., 2021; Chmielewski et al., 2021). They are also in training for professions where they will be working and interacting with people who experience various life situations and have different needs, behaviors, and reactions. Chen et al. (2021) suggested including mindfulness intervention in nursing education to promote the mental health of students.

Mindfulness-based stress reduction (MBSR) is a curriculum-based 8-week program that is designed to support participants in building resources to be present at the moment and to cope with stress and strains of life (Kabat-Zinn, 2005). These resources can also be expressed as a healthy non-judgmental attitude toward self, present-centered awareness, enhanced ability to regulate attention, stress, and emotions, and a greater prosocial attitude and behavior (Vago and Silbersweig, 2012). Several trials among various study populations have documented positive effects of MBSR on perceived stress, symptoms of anxiety and depression, and well-being (Khoury et al., 2015; de Vibe et al., 2017). However, Dimidjian and Segal have shown the lack of pragmatic trials that evaluate the effects of MBSR when implemented in real-life settings (effectiveness studies) (Dimidjian and Segal, 2015). Furthermore, evidence on mechanisms of change of MBSR is not well-established (Rosenkranz et al., 2019).

MBSR may be a sustainable program in addressing mental health challenges in students. Student teachers can be reached at their educational institution as suggested by the Perth Charter. Offering MBSR at educational institutions may both improve the mental health of the student teachers and their professional skills. Improving own mental health of student teachers, by providing them with mindfulness skills, might be a crucial first step in enhancing relational competencies, which are important in the teaching profession. Relational competence is the ability of the teacher to “see” the individual child on its own terms and attune its own behavior in the relation to the child to create the best possible environment for learning and developing (Juul and Jensen, 2017). When under pressure or in a period of stress, it can be difficult to be present and to attune an own behavior constructively. In the MBSR program, the students are e.g., trained to be present and to be aware of how they react when under pressure or in a stressful situation, and how it can be possible to respond in a more constructive way that is beneficial for creating good relationships (Peters et al., 2011).

In line with the Perth Charter (Clifford Beers Foundation and Mentally Health WA, 2012) and the third Sustainable Development Goal (Patel et al., 2018), a two-part project was offered to interested student teachers as an elective course at VIA University College in Aarhus, Denmark. The first part comprised the standardized 8-week MBSR program with an additional introduction day about the relevance of training own mental health as a student-teacher. The second part involved a total of 5 days, in total 20 h of training in relational competencies in regard to the teaching profession (Gøtzsche, 2018; Jensen and Gøtzsche, 2020). In the current study, we aimed to add to the research of effectiveness by investigating the effects of the mindfulness program (the first part of the two-part project) on the mental health of student teachers when offered at their educational institution in a real-life context. Furthermore, we aimed to explore some hypotheses of mechanisms of change.

We expected that MBSR would increase dispositional mindfulness and decrease mind wandering, which in turn would improve mental health in the students in terms of reducing stress, and symptoms of anxiety and depression as well as improving well-being and resilience.

## Methods

### Design and Setting

The study was designed as a parallel randomized waiting list-controlled trial (RCT) at VIA’s undergraduate program for teacher education in Aarhus, Denmark. It constituted a two-part project comprised of a mindfulness program including the standardized MBSR program and further training in relational competencies.

### Procedure

In August 2019 and August 2020, we informed about the research project and offered participation via communication channels at the institution, e.g., VIA’s intranet, the student newsletter, student emails, and joint student meetings. We informed that MBSR was an evidence-based program addressing mental health, and how to use mindfulness as a way of building relational competence in a professional context. We also presented an overview of the entire program. Furthermore, we gave information about the design of the research; that they would be randomly allocated either to an intervention group that would start the program immediately after randomization- or to a waiting list control group that would start the program the following semester. We also informed them; they would be asked to complete questionnaires for the research.

Approximately 2,800 student teachers could in principle have applied, but the program was offered as an extra-curricular course and it was impossible to avoid that some students had other courses or school placement interfering. Among the prospect population, 98 students showed interest in participating. To reflect real-life in this pragmatic trial design, usual procedures for MBSR participants were followed, and students were not recommended to participate in the project if they had (1) acute treatment-demanding clinical depression or a diagnosis of psychosis or schizophrenia, and/or (2) abuse of alcohol, drugs, and/or medicine. No students were, however, excluded based on these criteria, but some interested students could not participate for practical and logistical reasons. Interested students had to fill in an application with a personal description of themselves and motivation for participation. To confirm interest in participating in the research project, the students were invited to fill in the baseline questionnaire via a link sent by e-mail. A total of 67 self-selected students completed the baseline questionnaire and were randomized 1:1 to the intervention or the waiting list control group.

Randomization was conducted in two blocks with, respectively, 40 and 27 participants in September 2019 and August 2020. It was performed by a Statistician, who received by e-mail a list of the participants with an anonymous id concealing the true identity of the students. First, the participants were selected sorted randomly, and then allocated to the intervention or waiting list control group with an even sex distribution balance between the groups. The Statistician send by e-mail this anonymous allocation list with anonymous ids to the first author, who linked this to the true identity of the participants, creating the final allocation list. We informed the students by e-mail about their allocation. The intervention program started 2 weeks after randomization, at the beginning of the semester, and the waiting list program was in the following semester. This counts for both block randomizations. Hence, the first block intervention was delivered between September 14 and November 21, 2019, i.e., before the onset of coronavirus disease 2019 (COVID-19), in one group with 17 out of 20 allocated participants. The second block intervention was delivered in one group with 13 out of 14 allocated participants between August 29 and November 21, 2020. This was after the onset of COVID-19. Fortunately, there were no social distancing restrictions in autumn of 2020 in Denmark that prevented face-to-face teaching at the teacher education college. Hence, all interventions took place face-to-face at the teacher education college. The introduction day was taught by a teacher from VIA University College and a MBSR teacher, and the standardized MBSR curriculum (Kabat-Zinn, 2005; Mindfulness Center at Brown, 2020)^1^ was taught by a MBSR teacher that is trained from the Danish Center for Mindfulness. She had two different co-teachers, also MBSR teachers trained from Danish Center for Mindfulness, at the two MBSR courses. Danish Center for Mindfulness, Aarhus University is affiliated with Mindfulness Center, Brown University, and educates MBSR teachers according to international standards. The teachers were not members of the research group. They received supervision from a MBSR teacher from the Danish Center for Mindfulness in order to secure MBSR fidelity.

Participants allocated to the waiting list control group were offered the mindfulness program in the winter of 2020 and 2021.

### Intervention Content

The students that were allocated to the intervention group were offered the standardized MBSR program, which was preceded by a 6-h introduction day about the two-part project. The purpose of the introduction day was to introduce the students to the topic of relational competencies and how mindfulness/MBSR may be used in building capacity for relational competencies. Moreover, the students had the opportunity to get to know each other. The content of the day included small theoretical presentations on relational competencies and why relations are important when working with children. Furthermore, the day consisted of practical training; small mindfulness exercises on body and breath, dyad exercises on being aware of oneself and the other, and reflective group exercises.

MBSR is a curriculum-based program. It consists of 2.5-h weekly group sessions during 8 weeks, with one 7-h silent retreat day and 45–60 min of daily homework 6 days a week (Kabat-Zinn, 2005; Mindfulness Center at Brown, 2020; see text footnote 1). Every session has a specific theme such as perception, pleasant and unpleasant experiences, stress, and communication. The program includes meditation practices; body scan, yoga, sitting, and walking meditation.

### Blinding

Owing to the nature of the intervention, participants and the MBSR teachers were aware of group allocation for the duration of the study. The MBSR teachers were not involved in data collection. The team members involved in data collection and analysis were not blinded to group assignment.

### Data Collection and Management

We collected and stored questionnaire data at baseline and 3 months after baseline using the Research Electronic Data Capture (REDCap) tool hosted by Aarhus University. REDCap is a secure, web-based application designed to support data capture for research (Harris et al., 2009). We e-mailed two reminders in case of not responding to the follow-up questionnaire.

### Outcomes

#### Primary Outcome

The Perceived Stress Scale (PSS) is a measure of subjective stress. It consists of 10 items indicating how often respondents have experienced their lives as unpredictable, uncontrollable, and overloaded in the past month (Cohen et al., 1983). The items are scored on a five-point Likert scale (total sum scores: 0–40), and higher scores indicate higher levels of stress. The scale has demonstrated good validity and reliability (Cohen, 1988; Lee, 2012; Anita et al., 2015). The scale has been translated and validated in Danish context (Eskildsen et al., 2015), and Danish researchers have found higher scores of PSS associated with long-term sick leave (Larsen et al., 2018); use of primary health care (Prior et al., 2018); and mortality (Prior et al., 2016). Cronbach’s α was 0.86 in the present study sample. In a population-based Danish health profile from 2017, the mean PSS in the total population was 12.2, and it was respectively 14.2, 13.1, and 13.7 in the groups of 16–24-year-olds, women, and students (Sørensen et al., 2021).

#### Secondary Outcomes

##### Mental Health

The Hopkins Symptom Check List-5 (SCL-5) is a five-item self-report measure of symptoms of anxiety and depression (Tambs and Moum, 1993). All items are scored on a four-point scale, ranging from 1 (not bothered at all) to 4 (extremely bothered). The score is calculated as the average of the five items with higher scores indicating greater symptoms of anxiety and depression. It originates from the 90-item SCL, which has been translated and validated in a Danish context (Olsen et al., 2004). The SCL-5 correlates at *r* = 0.92 with the 25-item SCL- anxiety and depression subscale (Strand et al., 2003). A SCL-5 score >2 has been found to predict mental illness as assessed independently by psychiatrists (Strand et al., 2003). Cronbach’s α was 0.79 in the present study sample.

The WHO-5 Well-being Index (WHO-5) is a five-item self-report measure of well-being. It consists of five questions indicating the extent to which respondents have been feeling well during the last 2 weeks. Each question is scored on a five-point scale. The points are added and multiplied by four, calculating the total score ranging from 0 to 100; higher scores indicate higher levels of well-being. The WHO-5 has been translated into Danish (National Board of Health, 2021)^2^, and is considered to be a valid measure of the overall well-being of respondents (Topp et al., 2015). A score below 50 is indicative of mental health problems (Bech et al., 2003). Cronbach’s α was 0.81 in the present study sample.

The Brief Resilience Scale (BRS) is a six-item self-report measure of resilience (Smith et al., 2008). All items range from 1-5 (total sum range 6–30). The summary score is calculated as an average across the six items (range 1–5), with higher scores indicating a greater perceived recovery from stress (Smith et al., 2008). Cut-off points in scores have been suggested; low resilience: 1–2.99; normal resilience: 3–4.3; and high resilience: 4.31–5 (Smith et al., 2008). Cronbach’s α was 0.90 in the present study sample. Researchers from Danish Center for Mindfulness have translated BRS into Danish according to the WHO guideline including forward- and expert panel back-translation (CourseHero, 2021)^3^. Construct validation of BRS has not been performed in a Danish context. However, previous Danish research studies have shown the effect of MBSR on BRS (Juul et al., 2020; Diachenko et al., 2021).

##### Mindfulness Skills—Mediator Outcomes

The Five Facet Mindfulness Questionnaire (FFMQ-15) is a 15-item self-report measure of the dispositional tendency to be mindful in daily life (Gu et al., 2016). It is developed from the original FFMQ-39 and has been found to be reliable and valid (Gu et al., 2016). It includes five facets of mindfulness: Observing, Describing, Acting with awareness, Non-judgment, and Non-reactivity. Items are scored on a five-point scale. A total score is calculated by summing the scores of each sub-scale and then summing the sub-scores into one total score. Gu et al. (2016) has suggested that the subscore for the facet “observing” be omitted when calculating the total score. This was also found by a Danish research group, which translated and validated FFMQ in a Danish context (Jensen et al., 2019). In our study sample Cronbach’s α was 0.6 for Observing; 0.87 for Describing; 0.86 for Acting with awareness; 0.68 for Non-judgment; 0.72 for Non-reactivity; and finally 0.80 for the total score.

The Amsterdam Resting-State Questionnaire (ARSQ) is a self-report questionnaire, which samples thoughts and feelings during rest (i.e., an awake state characterized by the absence of goal-directed cognitive activity). The questionnaire consists of 21 statements scored on a Likert scale from 1 (completely disagree) to 5 (completely agree) after 5-min eyes-closed rest (Diaz et al., 2013). The ARSQ identifies seven dimensions of resting-state cognition: Discontinuity of Mind, Theory of Mind, Self, Planning, Sleepiness, Comfort, and Somatic Awareness. During the resting state, the mind typically wanders in a way that represents habitual ways of thinking. MBSR targets those habitual, normal, and persistent patterns of thoughts and feelings, hence the program has the potential to induce change. In the present study sample, Cronbach’s α was 0.71 for Discontinuity of Mind; 0.73 for Theory of Mind; 0.69 for Self; 0.79 for Planning; 0.73 for Sleepiness; 0.74 for Comfort; and 0.77 for Somatic Awareness. Researchers from Danish Center for Mindfulness have translated ARSQ into Danish according to the WHO guidelines including forward- and expert panel back-translation (CourseHero, 2021; see text footnote 3). ARSQ has not been validated in a Danish context. However, previous Danish research studies have shown the effect of MBSR on the ARSQ dimensions of discontinuity of mind, planning, body awareness, and comfort (Juul et al., 2020; Diachenko et al., 2021).

### Statistical Methods

We analyzed the data according to the intention-to-treat principle (ITT), i.e., all available data from participants were analyzed according to the randomization group the participants were originally assigned, regardless of what intervention they received (e.g., whether the participants in the intervention group completed the intervention or not). We used mixed-effect linear regression models with the fixed effects; randomization, sex, age, semester, time, intervention, and interaction between time and intervention. Each subject as well as the time of block randomization were specified as random effects in the model. The latter adjusted for the cluster effects regarding the MBSR course and time for implementation before or during the COVID-19 pandemic. The model can be expressed by the following equation:


$$Y=\beta_{0}+\beta_{T}\left(T=2\right)+\beta_{I}\left(I=1\right)+\beta_{I*T}\left(T=2\right)\left(I=1\right)+\beta_{M}\left(Sex=Male\right)+\beta_{A}\left(Age\right)+\beta_{S2}\left(S=2\right)+\beta_{S3}\left(S=3\right)+\beta_{S4}\left(S=4\right)+\beta_{S5}\left(S=5\right)+\beta_{S6}\left(S=6\right)+\beta_{S7}\left(S=7\right)+A_{Block}+B_{Id}+E_{Id*Time}$$

$$\mathrm{The}\mathrm{effect}=\beta_{I*T}$$

Moreover, we estimated Cohen’s d for all the outcomes by dividing the effect estimate by the pooled standard deviation (SD):


$$\sqrt{SD_{Interventiongroup}^{2}+SD_{Waitinglistcontrolgroup}^{2}}.$$

We used the following cut-points for the interpretation of the Cohen’s d results: 0.2: small effects, 0.5: medium effects, and 0.8: large effects (Lakens, 2013).

To take account of missing data, we conducted sensitivity analyses representing 4 scenarios with data not missing at random. Missing outcomes were substituted with the model-based prediction adding or subtracting 0.2 *SD* in the intervention or waiting list control arm. We also performed a loss to follow-up analysis regarding the primary outcome for sex, age, baseline PSS, SCL-5, WHO-5, and BRS by t-tests and chi_2_-tests. In order to justify combining and analyzing the two randomization blocks, which had been recruited before and after the onset of COVID-19, we compared the baseline mental health outcomes by *t*-tests.

**FIGURE 1:**
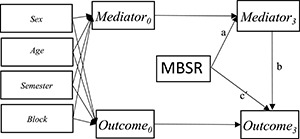
Autoregressive mediation model showing the a, b and c’ path.

In order to explore potential mediating effects, we used autoregressive models with a single mediator as depicted in Figure 1. We fitted the models in the SEM framework that, e.g., enable to analyze the action and the conceptual theory simultaneously. In statistical mediation analysis, the relationship between the intervention and the mediator is termed the *a* path. The conceptual theory; the association between the mediator and the outcome is termed the *b* path. The mediated effects were calculated by multiplying the *a* and *b* path coefficients. We calculated the 95% CI of the estimates of the mediated effects, by use of 50 bootstrap replications. The goodness of fit of the models was tested by the chi-squared test, the comparative fit index (CFI), and the root means squared error of approximation (RMSEA). We used the following criteria to evaluate model fit, wherein a CFI above 0.9 indicates a good model fit. A RMSEA below 0.08 indicates an acceptable model fit, and a RMSEA below 0.05 indicates a good model fit (Goldsmith et al., 2017; Loehlin and Beaujean, 2017).

We did not conduct power analyses. The (un)certainty of the results can be seen in the CIs. We present estimates with 95% CIs and *p*-values. We considered *p* < 0.05 as statistically significant.

We used the statistical package STATA 16 for all the analyses.

## Results

**FIGURE 2:**
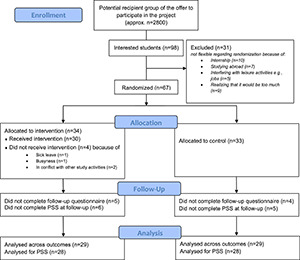
Flowchart.

Figure 2 shows the flowchart. A total of 67 students were recruited to the trial between August 23, 2019, to August 21, 2020. Follow-up data were collected between November 22, 2019, and January 12, 2021. The median follow-up time was 93 days in both intervention and waiting list control groups (q1 was 91 in both groups, and q3 was 101 in the intervention group and 99 in the waiting list control group).

Table 1 shows baseline characteristics. The median age was 25 years in both groups. The majority of the participants were women; 71% were in the intervention group, and 76% were in the waiting list control group. In the intervention group, 42% of the participants were students in the final 2 years of the undergraduate program for teacher education whereas the proportion was 71% of the participants in the waiting list control group.

**TABLE 1:** Characteristics of participants included in an RCT evaluating the effects of a mindfulness program including MBSR among students at VIA’s undergraduate program for teacher education (*n* = 67), Denmark, 2019 and 2020.

	**Mindfulness program (*n* = 34)**	**Waiting list (*n* = 33)**
Sex, female (%)	24 (71)	25 (76)
Age		
Median (q1,q3)	25.5 (24.0, 27.3)	25.4 (24.0, 27.2)
Mean (SD)	26.0 (2.9)	26.7 (5.1)
Semester (%)^a^		
1.	2 (6)	1 (3)
2.	1 (3)	1 (3)
3.	14 (45)	5 (16)
4.	1 (3)	2 (6)
5.	6 (19)	9 (29)
6.	0 (0)	1 (3)
7.	7 (23)	12 (39)

*RCT, Randomized controlled trial; MBSR, Mindfulness-Based Stress Reduction; q; quartile.*

*^a^Missing information: Intervention group, n = 3; Waiting list control group, n = 2.*

Table 2 shows mental health outcome scores at baseline and follow-up 3 months from baseline in both groups. At baseline, the self-reported mental health scores of the students indicated high levels of stress as well as symptoms of anxiety and depression in both groups; the mean PSS-score of the intervention group was 18.88 (*SD*: 7.75) and their mean SCL-5-score was 2.34 (*SD*: 0.6), while the mean PSS-score of the waiting list control group was 17.91 (*SD*: 6.36) and their mean SCL-5-score was 2.12 (*SD*: 0.44). Furthermore, the baseline measurements indicated levels of well-being within the normal range above the cut-point of 50 on the WHO-5 in both groups. Specifically, the intervention group showed a mean of 54.35 (*SD*: 17.31) and for the waiting list control, the mean was 60.36 (SD: 17.39). The levels of resilience were also within the normal range in both groups; the BRS-score was 3.59 (*SD*: 1.07) in the intervention group and 3.72 (*SD*: 0.84) in the waiting list control group. Supplementary Table 1 shows the baseline mental health scores for the two randomization blocks, which were recruited before and after the onset of COVID-19. There were no statistically significant differences between the blocks. The loss to follow-up in the primary outcome was non-differential, of which 6 (18%) in the intervention group and 5 (15%) in the waiting list control group. We found no specific characteristics associated with loss to follow-up (cf. Supplementary Table 2).

**TABLE 2:** Effects of a mindfulness program including MBSR on mental health among students at VIA’s undergraduate program for teacher education (*n* = 67) 3 months from baseline, Denmark, 2019 and 2020.

	**Mindfulness program**				**Control**				
		**Score**	**Within-group change from baseline**		**Score**	**Within-group change from baseline**	**Between-group difference**		
**Outcome**	**n**	**Mean (SD)**	**Mean (95% CI)^a^**	**n**	**Mean (SD)**	**Mean (95% CI)^a^**	**Mean (95% CI)^a^**	***p*-value**	**Cohens d**
**Perceived Stress Scale (PSS)**									
Baseline	34	18.88 (5.75)		32	17.91 (6.36)				
Follow-up	28	15.82 (6.39)	−3.25 (−5.52 to −0.98)	28	19.89 (5.95)	1.95 (−0.29 to 4.19)	−**5.20 (**−**8.39 to**−**2.01)**	**0.001**	**0.86**
**Symptom checklist- 5 (SCL-5)**									
Baseline	34	2.34 (0.60)		33	2.12 (0.44)				
Follow-up	28	2.13 (0.69)	−0.23 (−0.46 to −0.00)	27	2.40 (0.62)	0.29 (0.07 to 0.52)	−**0.52 (**−**0.84 to**−**0.20)**	**0.001**	**0.99**
**WHO-5 well-being Index (WHO-5)**									
Baseline	34	54.35 (17.31)		33	60.36 (17.39)				
Follow-up	28	57.57 (16.16)	3.73 (−3.90 to 11.37)	28	47.29 (18.41)	−13.21 (−20.67 to −5.75)	**16.94 (6.27 to 27.62)**	**0.002**	**0.98**
**Brief resilience scale, (BRS)**									
Baseline	34	3.59 (1.07)		33	3.72 (0.84)				
Follow-up	28	3.74 (1.01)	0.19 (−0.08 to 0.46)	28	3.72 (0.79)	0.08 (−0.19 to 0.34)	**0.11 (**−**0.27 to 0.49)**	**0.568**	**0.11**

*MBSR, Mindfulness-Based Stress Reduction.*

*^a^Adjusted for sex, age, semester, and cluster effect regarding randomization block (MBSR course and time) in mixed-effect linear regression models (described in more details under Statistical methods). Bold values effect estimates.*

A total of four students from the intervention group did not participate in any of the MBSR sessions (three from the first block randomization and one from the last). They all completed the follow-up questionnaire. Among the 30 students from the intervention group, who chose to start the MBSR program, the median number of sessions attended was 8 (q1:6, q3:9) out of 9 possible.

Table 2 further shows within-group changes and between-group differences in the mental health outcomes as well as Cohen’s d estimates for the latter. The students in the intervention group improved their mental health during the 3 months of follow-up, whereas the mental health decreased among the students in the waiting list control group. We found a statistically significant effect of the mindfulness program on the primary outcome PSS, –5.2 (95% CI –8.39 to –2.01) with a corresponding Cohen’s d 0.86. We also found statistically significant effects of the mindfulness program on the secondary mental health outcomes SCL-5, –0.52 (95% CI –0.84 to –0.2), Cohen’s d 0.99, and WHO-5, 16.94 (95% CI 6.27–27.62), Cohens d 0.98. We found no effect on BRS, 0.11 (95% CI –0.27 to 0.49), Cohen’s d 0.11.

Table 3 shows the results regarding the mindfulness skills, the mediator outcomes. The ARSQ measurement indicated effects of the mindfulness program on distracting thoughts, thoughts of self and comfort in resting-state; Discontinuity of mind: –2.05 (−3.48 to –0.63), Cohen’s d 0.78; Self: −1.68 (−3.18 to –0.19), Cohens d 0.68; Comfort: 2.24 (0.84–3.63), Cohens d 1.03. We found no effect on the other ARSQ dimensions or the FFMQ subscales or total score.

**TABLE 3:** Effects of a mindfulness program including MBSR on mediator outcomes among students at VIA’s undergraduate program for teacher education (*n* = 67) 3 months from baseline, Denmark, 2019 and 2020.

	**Mindfulness program**				**Control**				
		**Score**	**Within-group change from baseline**		**Score**	**Within-group change from baseline**	**Between-group difference**		
**Outcome**	**n**	**Mean (SD)**	**Mean (95% CI)^a^**	**n**	**Mean (SD)**	**Mean (95% CI)^a^**	**Mean (95% CI)^a^**	***p*-value**	**COHENS d**
**Five Facet Mindfulness Questionairre-15 (FFMQ)**									
Observing									
Baseline	34	9.26 (1.93)		33	9.85 (3.14)				
Follow-up	28	10.04 (1.50)	0.66 (−0.15 to 1.47)	27	10.07 (2.63)	0.38 (−0.43 to 1.18)	**0.28 (**−**0.86 to 1.42)**	**0.628**	**0.11**
Describing									
Baseline	34	10.41 (2.24)		33	11.18 (2.58)				
Follow-up	28	10.86 (2.41)	0.56 (−0.22 to 1.34)	28	10.75 (3.31)	−0.27 (−1.03 to 0.49)	**0.83 (**−**0.26 to 1.92)**	**0.134**	**0.34**
Acting with awareness									
Baseline	34	8.94 (2.66)		33	9.61 (2.77)				
Follow-up	28	9.54 (1.93)	0.76 (−0.05 to 1.56)	28	9.75 (2.32)	0.05 (−0.73 to 0.84)	**0.71 (**−**0.42 to 1.83)**	**0.218**	**0.26**
Non-judging of inner experience									
Baseline	34	9.82 (2.46)		33	9.76 (3.05)				
Follow-up	27	9.85 (2.82)	0.14 (−0.81 to 1.09)	28	9.75 (2.86)	−0.44 (−1.35 to 0.47)	**0.58 (**−**0.74 to 1.90)**	**0.388**	**0.21**
Non-reactivity to inner experience									
Baseline	34	7.94 (1.92)		32	8.09 (2.02)				
Follow-up	28	8.75 (1.84)	0.86 (0.04 to 1.68)	28	8.39 (2.15)	0.28 (−0.53 to 1.10)	**0.58 (**−**0.58 to 1.73)**	**0.328**	**0.29**
Total score									
Baseline	34	37.12 (5.62)		32	38.34 (6.93)				
Follow-up	28	39.15 (6.74)	2.17 (−0.01 to 4.36)	27	38.64 (7.06)	−0.35 (−2.47 to 1.77)	**2.52 (**−**0.53 to 4.36)**	**0.105**	**0.40**
**Amsterdam Resting State Questionnaire (ARSQ)**									
Discontinuity of mind									
Baseline	34	9.62 (2.89)		33	10.39 (2.33)				
Follow-up	27	8.15 (2.46)	−2.09 (−3.12 to −1.05)	28	10.25 (3.11)	−0.03 (−1.02 to 0.95)	−**2.05 (**−**3.48 to**−**0.63)**	**0.005**	**0.78**
Theory of mind									
Baseline	34	9.03 (2.78)		32	9.66 (2.40)				
Follow-up	27	8.11(2.93)	−1.01 (−2.30 to 0.27)	28	9.61(2.28)	0.03 (−1.22 to 1.28)	−**1.05 (**−**2.84 to 0.75)**	**0.253**	**0.40**
Self									
Baseline	33	10.27 (2.54)		33	10.73 (2.39)				
Follow-up	28	8.82 (2.34)	−1.61 (−2.68 to −0.53)	28	10.71 (3.00)	0.08 (−0.96 to 1.11)	−**1.68 (**−**3.18 to**−**0.19)**	**0.027**	**0.68**
Planning									
Baseline	34	10.65 (3.28)		32	11.56 (2.40)				
Follow-up	28	9.04 (3.43)	−1.59 (−3.01 to −0.16)	29	11.21 (2.29)	−0.32 (−1.71 to 1.07)	−**1.27 (**−**3.26 to 0.72)**	**0.211**	**0.44**
Sleepiness									
Baseline	34	6.68 (2.42)		33	6.91 (2.47)				
Follow-up	26	6.00 (2.74)	−0.45 (−1.65 to 0.74)	27	7.19 (2.37)	0.29 (−0.88 to 1.45)	−**0.74 (**−**2.41 to 0.93)**	**0.384**	**0.30**
Comfort									
Baseline	34	9.35 (2.27)		33	10.18 (2.08)				
Follow-up	28	10.18 (2.26)	0.97 (−0.03 to 1.96)	28	9.07 (2.60)	−1.27 (−2.24 to −0.30)	**2.24 (0.84 to 3.63)**	**0.002**	**1.03**
Somatic awareness									
Baseline	34	9.44 (2.87)		33	9.06 (3.04)				
Follow-up	28	11.04 (2.08)	1.07 (−0.13 to 2.26)	28	8.96 (2.52)	−0.07 (−1.23 to 1.09)	**1.14 (**−**0.53 to 2.80)**	**0.181**	**0.39**

*MBSR, Mindfulness Based Stress Reduction.*

*^a^Adjusted for sex, age, semester, and cluster effect regarding randomization block (MBSR course and time) in mixed-effect linear regression models (described in more details under Statistical methods). Bold values effect estimates.*

All the effects remained statistically significant in the sensitivity analyses (cf. Supplementary Tables 3, 4).

**TABLE 4:** Results of single mediation models^a^ in an RCT evaluating the effects of a mindfulness program including MBSR among students at VIA’s undergraduate program for teacher education (*n* = 67), Denmark.

**Outcome (OC)**		**Perceived stress scale**	**Symptom checklist- 5**	**WHO-5 well-being index**
		**Estimate (95% CI), p^b^**	**Estimate (95% CI), p^b^**	**Estimate (95% CI), p^b^**
**Mediator: Amsterdam Resting State Questionnaire (ARSQ)**				
**Discontinuity of mind (DOM)**				
*a* path coefficient	MBSR- > DOM	−2.01 (−3.31 to −0.71), 0.002	−2.07 (−3.37 to −0.76), 0.002	−2.02 (−3.32 to −0.72), 0.002
*b* path coefficient	DOM- > OC	0.85 (0.40 to 1.30), < 0.001	0.06 (0.01 to 0.11), 0.026	−2.01 (−3.48 to −0.54), 0.007
Mediated effect	MBSR- > DOM- > OC	−1.72 (−3.14 to −0.30), 0.018	−0.12 (−0.28 to 0.04). 0.131	4.07 (−0.69 to 8.83), 0.094
Goodness of fit				
(df) = X^2^		(14) = 16.82, 0.211	(14) = 20.2, 0.123	(14) = 20.96, 0.103
RMSEA		0.06	0.09	0.10
CFI		0.95	0.87	0.85
**Self**				
*a* path coefficient	MBSR- > SELF	−1.90 (−3.18 to −0.61), 0.004	−1.90 (−3.18 to −0.61), 0.004	−1.89 (−3.18 to −0.61), 0.002
*b* path coefficient	SELF- > OC	0.57 (0.08 to 1.06), 0.024	0.07 (0.02 to 0.13), 0.008	−1.19 (−2.82 to 0.44), 0.153
Mediated effects	MBSR- > SELF- > OC	−1.08 (−2.26 to 0.09), 0.071	−0.14 (−0.27 to −0.01), 0.037	2.25 (−1.69 to 6.19), 0.264
Goodness of fit				
(df) = X^2^, p		(14) = 19.41, 0.150	(14) = 34.9, 0.002	(14) = 29.05, 0.010
RMSEA		0.09	0.17	0.15
CFI		0.86	0.56	0.57
**Comfort**				
*a* path coefficient	MBSR- > COMFORT	1.58 (0.36 to 2.80), 0.011	1.57 (0.35 to 2.79), 0.011	1.55 (0.33 to 2.77), 0.013
*b* path coefficient	COMFORT- > OC	−1.06 (−1.58 to −0.54), < 0.001	−0.11 (−0.17 to −0.05), 0.001	3.50 (1.99 to 5.01), < 0.001
Mediated effects	MBSR- > COMFORT > OC	−1.68 (−3.35 to −0.01), 0.049	−0.17 (−0.36 to 0.02), 0.072	5.41 (−0.70 to 11.52), 0.083
Goodness of fit				
(df) = X^2^, p		(14) = 24.7, 0.037	(14) = 32.3, 0.004	(14) = 17.40, 0.236
RMSEA		0.12	0.16	0.07
CFI		0.77	0.58	0.89

*RCT, Randomized controlled trial; MBSR, Mindfulness Based Stress Reduction.*

*^a^According to Figure 1.*

*^b^Adjusted for sex, age, semester, randomization block using structural equation models (described in more details under Statistical methods).*

Table 4 shows the results of three mediation models each including a single mediator outcome of which we found statistically significant effects from the mindfulness program (ARSQ-Discontinuity of Mind, -Self, and -Comfort). We found statistically significant mediating effects of the mindfulness program via ARSQ-Discontinuity of mind on PSS: −1.72 (−3.14 to −0.3). We also found a statistically significant mediating effect via ARSQ-Self on SCL-5: −0.14 (−0.27 to −0.01). Finally, we found a statistically significant mediating effect via ARSQ-Comfort on PSS: −1.68 (−3.35 to −0.01). The goodness of fit tests showed only acceptable model fit for the ARSQ-Discontinuity of mind/PSS model. Regarding the mediator outcomes with no shown statistically significant effects from the mindfulness program in this study, the associations of the mediator outcomes with the mental health outcomes are presented in Supplementary Table 5 in terms of the *b* paths coefficients with 95% CI and *p*-values. These analyses showed that the FFMQ total score and especially the subscales Non-judging of and Non-reactivity to inner experience were statistically significantly associated with the mental health outcomes.

## Discussion

### Providing Access to a Mindfulness Program for Students Teachers at Their Educational Institution Has Large Effects on Mental Health and Seems Like a Beneficial Preventive Strategy

We found large effects on the mental health of student teachers in terms of stress reduction, reduction of symptoms of anxiety and depression, and improvement in well-being from offering a mindfulness program including the standardized MBSR program at their educational institution in a real-life context. The approach of providing access to effective health promotion interventions for young people in their daily lives is highly in line with recommendations from WHO and The Lancet Commission on global mental health and sustainable development (Clifford Beers Foundation and Mentally Health WA, 2012; Patel et al., 2018). Our findings of effect on mental health are consistent, even larger compared with other studies examining effects of MBPs for students. In comparison, systematic reviews and meta-analyses among university students (Dawson et al., 2020) and nursing students (Chen et al., 2021) found that MBPs improve mental health by decreasing distress and symptoms of anxiety and depression and improving well-being with small to moderate effects sizes. However, the content and duration of the included MBPs varied to a large degree. A study of Norwegian medical and psychology students attending an MBSR program at the beginning of their education showed effects on their mental health, stress, well-being, and mindfulness post-intervention (de Vibe et al., 2013). Follow-up results indicate lasting effects, as well as more desirable coping strategies in the intervention group compared to the control group 4 and 6 years after intervention (de Vibe et al., 2018; Solhaug et al., 2019). Beyond promoting health and coping strategies during the years of attending college and university, the long-term effects hold the potential to be of benefit in future professional challenges both at the individual and interpersonal level. This may impact their caregiving abilities, their resilience, and their resources to persist on the job. Also, regarding the teaching profession, the transmission from education to the profession can be very stressful. The theoretical learning about the profession can seem very far away when you are confronted with conflicts with children, parents, colleagues and at the same time has the responsibility for a class of children. Teacher stress and correlated lower engagement affect pupil engagement and cause lower achievements (Gordon, 2010). In addition, other consequences include unconducive learning and classroom environments (McLean and McDonald Connor, 2015) and greater behavior problems in the classroom. These issues relate to lower levels of social adjustments and academic performance (Hoglund et al., 2015), which in turn may impact teacher stress. In Denmark, teachers constitute a vulnerable group when it comes to experiencing high levels of stress in the job (National Research Centre for the Working Environment, 2018). The OECD Teaching and Learning International Survey (TALIS) claims that more than 50% of school teachers in Denmark report experiencing “a lot” or “quite a lot” of stress on the job (Danish Evaluation Institute, 2020). A large body of evidence has shown promising effects of MBPs on the stress, anxiety, depression, and well-being of teachers (Emerson et al., 2017; Hwang et al., 2017; Lomas et al., 2017; Klingbeil and Renshaw, 2018; Zarate et al., 2019). Our trial showing the effects of a mindfulness program in teacher students on their mental health suggests it is appropriate to embed mindfulness training already in the teacher education. Klingbeil and Renshaw also found indications of small effects on classroom climate of MBPs for teachers. Therefore potentially, providing student teachers mindfulness training may also be beneficial for their future life as a teacher and their teacher competencies.

### No Effects on Resilience or Mindfulness

Unexpectedly, our trial did not show the effect of the mindfulness program on resilience. No effect of MBPs on resilience is in line with findings from meta-analysis among university students (Dawson et al., 2020). Previous trials had shown the effect of MBSR on resilience. These trials included study populations at an older age and with lower levels of baseline resilience scores although within the normal range (Juul et al., 2020; Diachenko et al., 2021). It was also very unexpected that our trial did not show a statistically significant effect of the mindfulness program on dispositional mindfulness and thereby no mediating effects. In comparison, Dawson et al. (2020) and Chen et al. (2021) found moderate effects of MBPs on mindfulness in their meta-analyses among university—and nursing students. The confidence intervals of our results in Table 3 showed that the effect of the mindfulness program on mindfulness was associated with an uncertainty that the program could decrease the mindfulness score by 0.5 score-points or increase the mindfulness score by 4.4 score-points. Despite the uncertainty and maybe lack of statistical power, the confidence intervals indicate the highest potential impact of the mindfulness program on the mindfulness score to be 4.4 score-points in our trial context. The *b* path estimates in Supplementary Table 5 suggested that e.g., every 10 score-points increase in the mindfulness score were associated with an expected reduction in perceived stress of 3.8 score-points (95% CI 1.9–5.7); an expected reduction in symptoms of anxiety and depression of 0.4 score-points (95% CI 0.2–0.6); an expected increase in well-being of 10.3 score-points (95% CI 4.8–15.9); and an expected increase in resilience of 0.5 score-points (95% CI 0.2–0.7). Hence, the conceptual theory that dispositional mindfulness is associated with improved mental health is supported by our study. The action theory; the effect of the mindfulness program on dispositional mindfulness, on the other hand, was not supported in our trial, and our findings of the effects of the mindfulness program on mental health could not be explained by improvement in dispositional mindfulness.

### Reduced Distracting Thoughts; A Potential Mechanism of Change

On the other hand, our findings of positive effects of the mindfulness program on distracting thoughts, thoughts of self, and comfort in resting-state were in line with our hypotheses that the mindfulness program decreases mind wandering and increases a non-judgmental attitude e.g., toward self. Former trials had also shown the effect of MBSR on the resting state dimensions of discontinuity of mind and comfort (Juul et al., 2020; Diachenko et al., 2021). Killingworth and Gilbert showed that our human minds about half of our awake hours are occupied with mind wandering with consequences for our mental health (Killingsworth and Gilbert, 2010). They showed that paying attention in the present moment was associated with well-being despite the quality of activities in the present moment (Killingsworth and Gilbert, 2010). Our mediation analysis showed promising results that the effect of the mindfulness program on stress reduction was to some extent associated with and maybe could be partly explained by reduced distracting thoughts.

### Strengths

#### High External Validity

The study was designed as an RCT in a real-life setting of an educational institution providing high external validity. The participants constituted a self-selected group of students. Compared to the Danish background population, the self-selected student teachers participating in this RCT had mental health challenges at baseline based on self-reported outcomes (Sørensen et al., 2021). Their mean score on perceived stress indicated e.g., a higher risk for long-term sick leave by having a PSS score > 15 (Larsen et al., 2018). However, we presume that they are representative of the group of students who would sign up for the course if it became a permanent offering at educational institutions. A major strength of offering interventions as a population-based strategy is to avoid stigmatizing. Regarding teacher competencies and mental health in general, every student teacher could potentially benefit from the mindfulness program as it is a universal intervention. However, the self-selected students were characterized by experiencing mental health challenges. This could indicate that poor mental health is a motivating factor for enrolling in the program, making the intervention more preventive in nature. This observation supports the possibility that participation could prevent sick leave or dropping out of university among this self-selected group.

#### Manualized Intervention, High Intervention Participation Rate, and Low Risk of Selection Bias

It is also a strength that we applied the standardized MBSR program taught by well-educated MBSR teachers, and the participation rate was high. Furthermore, the loss to follow-up in the RCT was relatively small and non-differential with no specific measured characteristics of the students leaving the study, making the risk of selection bias low. This was supported by the results of a rigorous sensitivity analysis. According to national law, it is optional for the participants to complete the questionnaires. Therefore, we did not contact non-responders after the two reminders.

#### Low Risk of the Impact of COVID-19 on the Study Results

Parts of the trial were conducted after the onset of the COVID-19 pandemic, which potentially could affect intervention delivery and results. Fortunately, there were no social distancing restrictions in Denmark in the period of intervention delivery in autumn of 2020 that prevented face-to-face teaching at the teacher education college, and MBSR was taught face-to-face according to the curriculum. The baseline mental health outcome measures were comparable in the two randomization blocks, which were recruited before and after the onset of COVID-19. In our statistical analysis, we adjusted for cluster effect representing being part of a group before or during COVID-19.

#### High-Quality Statistical Analysis

We analyzed our data by use of appropriate and robust statistical models.

### Limitations

#### Risk of Information Bias and Short Follow-Up

We were not able to blind the participants for intervention allocation. Therefore, we cannot rule out the possibility that information bias was present as we only used self-reported outcomes. It was not possible to collect objective information on e.g., sick leave and dropout. Neither was it possible to collect further follow-up data as the study was designed with a waiting-list control group, which was promised the mindfulness program shortly after the intervention group finished their program.

#### Risk of Unspecific Effects

Limitations of this study also include that we did not directly assess MBSR teacher competencies and intervention fidelity—only indirectly by supervision. Neither, did we measure the amount of homework, i.e., meditation practice done by the students. Furthermore, we did not collect data on adverse events in this trial. It should also be noted that the intervention evaluated in this study also included the 6-h introductory day about relational competencies in education and teaching, which preceded the MBSR program. Hence, questions about specific and unspecific effects of the MBSR program are relevant and should be addressed in future studies designed for that purpose e.g., with active control groups and rigorously designed mediation studies. Our mediation analysis was limited due to several reasons. First of all, the trial only included two measurement points and thereby lack the requirement of temporal ordering. Secondly, only simple mediation models including a single mediator were analyzed. Finally, the confidence intervals for the estimates of mediated effects were wide, indicating a lack of statistical power in the mediation analysis. However, the results can provide some hypotheses about mechanisms of change of MBSR, which can be explored in future more well-powered studies.

## Conclusion

Our findings suggested that offering a mindfulness program that includes the standardized MBSR program at an undergraduate program for teacher education could significantly improve the mental health among self-selected students within 3 months. Results of mediation analysis supported the hypothesis that some of the effects might be explained by reduced distracting thoughts.

## Data Availability Statement

The raw data supporting the conclusions of this article will be made available by the authors, without undue reservation.

## Ethics Statement

Ethical review and approval was not required for the study on human participants in accordance with the local legislation and institutional requirements. Written informed consent for participation was not required for this study in accordance with the national legislation and the institutional requirements.

## Author Contributions

LJ, KG, BN, and LF designed the study. LJ collected, analyzed, and interpreted the data. LJ drafted the article, and EB provided valuable input to the first draft of the manuscript. KG, BN, and LF critically revised the article. All authors contributed to the article and approved the version submitted.

## Conflict of Interest

Aarhus University offers revenue-funded MBSR teacher training education and MBSR courses. The authors declare that the research was conducted in the absence of any commercial or financial relationships that could be construed as a potential conflict of interest.

## Publisher’s Note

All claims expressed in this article are solely those of the authors and do not necessarily represent those of their affiliated organizations, or those of the publisher, the editors and the reviewers. Any product that may be evaluated in this article, or claim that may be made by its manufacturer, is not guaranteed or endorsed by the publisher.

## Footnotes

1
https://www.brown.edu/public-health/mindfulness/ideas/good-practice-guidelines-teaching-mbsr

2
https://www.regionsjaelland.dk/Kampagner/broen-til-bedre-sundhed/Materialer/PublishingImages/WHO5%20skema.pdf

3
https://www.coursehero.com/file/30372721/WHO-Process-of-translation-and-adaptation-of-instrumentspdf/
