# Supplementary material for: The Effects of a Mindfulness Program on Mental Health in Students at an Undergraduate Program for Teacher Education: A Randomized Controlled Trial in Real-Life
Source: Front Psychol. 2021 Dec 6;12:722771. doi: 10.3389/fpsyg.2021.722771 (PMC8687132; doi:10.3389/fpsyg.2021.722771)
Supplement: Supplementary file 1 [file Table_1.DOCX]

| **Supplemental table 1.** Baseline scores on mental health outcomes in the two randomization blocks. Participants in  the first randomization block was recruited before the onset of COVID-19 and the second randomization block after  the onset. | | | |
| --- | --- | --- | --- |
|  | **Block 1**  **August 2019**  **(n=40)**  **Mean (SD)** | **Block 2**  **August 2020**  **(n=27)**  **Mean (SD)** | **P-value** |
| **Perceived Stress Scale** | 18.69 (5.98) | 18.00 (6.18) | 0.65 |
| **Symptom Checklist-5** | 2.23 (0.52) | 2.24 (0.57) | 0.96 |
| **WHO-5 Well-being Index** | 58.4 (18.7) | 55.7 (15.6) | 0.54 |
| **Brief Resilience Scale** | 3.52 (0.89) | 3.85 (1.03) | 0.17 |
|  |  |  |  |
|  |  |  |  |
