# Supplementary material for: The Effects of a Mindfulness Program on Mental Health in Students at an Undergraduate Program for Teacher Education: A Randomized Controlled Trial in Real-Life
Source: Front Psychol. 2021 Dec 6;12:722771. doi: 10.3389/fpsyg.2021.722771 (PMC8687132; doi:10.3389/fpsyg.2021.722771)
Supplement: Supplementary file 2 [file Table_2.DOCX]

**Supplemental table 2**. Loss to follow-up analysis (primary outcome, PSS) in a RCT evaluating effects of a mindfulness intervention among students at VIA's undergraduate program for teacher education (n=67), Denmark, 2019 and 2020.

| **Characteristics** | Analyzed  (n=56) | Lost to follow up (n=11) | p-value |
| --- | --- | --- | --- |
| Sex |  |  |  |
| Women, n (%) | 42 (75) | 7 (64) |  |
| Men, n (%) | 14 (25) | 4 (36) | 0.45 |
| Age, mean (SD) | 27 (4) | 25 (2) | 0.36 |
| Baseline mental health, mean (SD) |  |  |  |
| Perceived Stress Scale | 18.69 (6.20) | 17.00 (5.04) | 0.40 |
| Symptom Check List-5 | 2.23 (0.51) | 2.25 (0.65) | 0.88 |
| WHO-5 Well-being Index | 57.07 (18.03) | 58.55 (15.10) | 0.80 |
| Brief Resilience Scale | 3.57 (0.97) | 4.02 (0.78) | 0.11 |
