# Supplementary material for: The Effects of a Mindfulness Program on Mental Health in Students at an Undergraduate Program for Teacher Education: A Randomized Controlled Trial in Real-Life
Source: Front Psychol. 2021 Dec 6;12:722771. doi: 10.3389/fpsyg.2021.722771 (PMC8687132; doi:10.3389/fpsyg.2021.722771)
Supplement: Supplementary file 3 [file Table_3.DOCX]

| **Supplemental table 3**. Sensitivity analysis regarding mental health outcomes | | | | | | | | |
| --- | --- | --- | --- | --- | --- | --- | --- | --- |
|  | **MBSR intervention** | | |  | | **Wait-list control** | |  |
|  | Between-group difference | 95% CI | | p-value | | Between-group difference | 95% CI | p-value |
| **Primary outcome** | | | | | | | | |
| **+ 0.2 SD*** | | | | | | | | |
| **PSS** | | | | | | | | |
| Follow-up | -4.81 | -7.71 to -1.90 | 0.001 | | -5.09 | | -7.98 to -2.19 | 0.001 |
| **- 0.2 SD*** | | | | | | | | |
| **PSS** | | | | | | | | |
| Follow-up | -5.21 | -8.07 to -2.35 | <0.001 | | -4.93 | | -7.80 to -2.05 | 0.001 |
| **Secondary mental health outcomes** | | | | | | | | |
| **+ 0.2 SD*** | | | | | | | | |
| **SCL-5** | | | | | | | | |
| Follow-up | -0.48 | -0.77 to -0.19 | <0.001 | | -0.52 | | -0.81 to -0.23 | <0.001 |
| **WHO-5** | | | | | | | | |
| Follow-up | 17.50 | 7.74 to 27.24 | <0.001 | | 16.58 | | 6.83 to 26.32 | 0.001 |
| **BRS** |  | - |  | |  | |  |  |
| Follow-up | 0.14 | -0.22 to 0.49 | 0.450 | | 0.09 | | -0.26 to 0.44 | 0.626 |
| **- 0.2 SD*** | | | | | | | | |
| **SCL-5** | | | | | | | | |
| Follow-up | -0.52 | -0.81 to -0.23 | <0.001 | | -0.49 | | -0.78 to -0.20 | 0.001 |
| **WHO-5** | | | | | | | | |
| Follow-up | 16.35 | 6.55 to 26.14 | 0.001 | | 17.27 | | 7.48 to 27.06 | 0.001 |
| **BRS** |  |  |  | |  | |  |  |
| Follow-up | 0.08 | -0.28 to 0.43 | 0.682 | | 0.12 | | -0.24 to 0.48 | 0.502 |
| * Missing values were replaced by model-based predictions adding or subtracting 0.2 SD to predicted in either the intervention or the wait-list control arm. Thus, when adding or subtracting 0.2 SD to the predicted in the intervention group, the wait-list control group estimate was kept as predicted. When adding or subtracting 0.2 SD to predicted in the wait-list control group, the intervention group estimate was kept as predicted.  Abbreviations: CI: confidence interval, MBSR: Mindfulness-based Stress Reduction, PSS: Perceived Stress Scale, SCL-5: The Hopkins Symptom Checklist-5, SD: standard deviation, WHO-5: The WHO-5 Well-being Scale; BRS: Brief Resilience Scale | | | | | | | | |
