# Supplementary material for: The Effects of a Mindfulness Program on Mental Health in Students at an Undergraduate Program for Teacher Education: A Randomized Controlled Trial in Real-Life
Source: Front Psychol. 2021 Dec 6;12:722771. doi: 10.3389/fpsyg.2021.722771 (PMC8687132; doi:10.3389/fpsyg.2021.722771)
Supplement: Supplementary file 4 [file Table_4.DOCX]

| **Supplemental table 4.** Sensitivity analysis regarding mediator outcomes | | | | | | | | |
| --- | --- | --- | --- | --- | --- | --- | --- | --- |
|  | **MBSR intervention** | | |  | | **Wait-list control** | |  |
|  | Between-group difference | 95% CI | | p-value | | Between-group difference | 95% CI | p-value |
| **+ 0.2 SD*** | | | | | | | | |
| **Five Facet Mindfulness Questionnaire** | | | | | | | | |
| **Observing** | | | | | | | | |
| Follow-up | 0.46 | -0.62 to 1.53 | 0.41 | | 0.32 | | -0.75 to 1.38 | 0.58 |
| **Describing** |  |  |  | |  | |  |  |
| Follow-up | 0.86 | -0.15 to 1.86 | 0.10 | | 0.72 | | -0.29 to 1.73 | 0.16 |
| **Acting with awareness** |  |  |  | |  | |  |  |
| Follow-up | 0.60 | -0.42 to 1.63 | 0.25 | | 0.48 | | -0.56 to 1.52 | 0.37 |
| **Non-judging of inner experience** |  |  |  | |  | |  |  |
| Follow-up | 0.62 | -0.60 to 1.84 | 0.32 | | 0.46 | | -0.76 to 1.69 | 0.46 |
| **Non-reactivity to inner experience** |  |  |  | |  | |  |  |
| Follow-up | 0.65 | -0.39 to 1.69 | 0.22 | | 0.56 | | -0.48 to 1.60 | 0.29 |
| **Total score** |  |  |  | |  | |  |  |
| Follow-up | 2.57 | -0.24 to 5.39 | 0.07 | | 2.23 | | -0.76 to 4.89 | 0.15 |
| **Amsterdam Resting State Questionnaire** |  |  |  | |  | |  |  |
| **Discontinuity of mind** |  |  |  | |  | |  |  |
| Follow-up | -1.63 | -2.99 to -0.26 | 0.02 | | -1.79 | | -3.13 to -0.45 | 0.01 |
| **Theory of mind** |  |  |  | |  | |  |  |
| Follow-up | -0.97 | -2.59 to 0.64 | 0.24 | | -1.11 | | -2.73 to 0.51 | 0.18 |
| **Self** |  |  |  | |  | |  |  |
| Follow-up | -1.49 | -2.93 to -0.05 | 0.02 | | -1.61 | | -3.03 to -0.22 | 0.02 |
| **Planning** |  |  |  | |  | |  |  |
| Follow-up | -1.13 | -3.00 to 0.74 | 0.24 | | -1.25 | | -3.10 to 0.61 | 0.19 |
| **Sleepiness** |  |  |  | |  | |  |  |
| Follow-up | -0.60 | -2.12 to 0.92 | 0.44 | | -0.76 | | -2.27 to 0.74 | 0.32 |
| **Comfort** |  |  |  | |  | |  |  |
| Follow-up | 2.22 | 0.93 to 3.52 | 0.001 | | 2.10 | | 0.80 to 3.41 | 0.002 |
| **Somatic awareness** |  |  |  | |  | |  |  |
| Follow-up | 1.27 | -0.32 to 2.86 | 0.12 | | 1.13 | | -0.46 to 2.71 | 0.17 |
| **Supplemental Table 2.** Sensitivity analysis regarding mediator outcomes, continued… | | | | | | | | |
|  | **MBSR intervention** | | |  | | **Wait-list control** | |  |
|  | Between-group difference | 95% CI | | p-value | | Between-group difference | 95% CI | p-value |
| **- 0.2 SD*** | | | | | | | | |
| **Five Facet Mindfulness Questionnaire** | | | | | | | | |
| **Observing** | | | | | | | | |
| Follow-up | 0.30 | -0.77 to 1.37 | 0.58 | | 0.44 | | -0.64 to 1.52 | 0.56 |
| **Describing** |  |  |  | |  | |  |  |
| Follow-up | 0.69 | -0.35 to 1.72 | 0.19 | | 0.82 | | -0.21 to 1.85 | 0.12 |
| **Acting with awareness** |  |  |  | |  | |  |  |
| Follow-up | 0.45 | 0.60 to 1.50 | 0.41 | | 0.57 | | -0.46 to 1.61 | 0.28 |
| **Non-judging of inner experience** |  |  |  | |  | |  |  |
| Follow-up | 0.41 | -0.81 to 1.63 | 0.51 | | 0.57 | | -0.64 to 1.78 | 0.36 |
| **Non-reactivity to inner experience** |  |  |  | |  | |  |  |
| Follow-up | 0.52 | -0.52 to 1.56 | 0.32 | | 0.61 | | -0.43 to 1.65 | 0.29 |
| **Total score** |  |  |  | |  | |  |  |
| Follow-up | 2.07 | -0.76 to 4.89 | 0.15 | | 2.40 | | -0.42 to 5.23 | 0.10 |
| **Amsterdam Resting State Questionnaire** |  |  |  | |  | |  |  |
| **Discontinuity of mind** |  |  |  | |  | |  |  |
| Follow-up | -1.84 | 3.17 to -0.52 | 0.01 | | -1.68 | | -3.03 to -0.33 | 0.02 |
| **Theory of mind** |  |  |  | |  | |  |  |
| Follow-up | -1.18 | -2.81 to 0.46 | 0.16 | | -1.04 | | -2.66 to 0.58 | 0.21 |
| **Self** |  |  |  | |  | |  |  |
| Follow-up | -1.63 | -3.03 to -0.22 | 0.02 | | -1.51 | | -2.93 to -0.09 | 0.04 |
| **Planning** |  |  |  | |  | |  |  |
| Follow-up | -1.33 | -3.16 to 0.51 | 0.16 | | -1.21 | | -3.06 to 0.64 | 0.20 |
| **Sleepiness** |  |  |  | |  | |  |  |
| Follow-up | -0.79 | -2.31 to 0.72 | 0.31 | | -0.63 | | -2.16 to 0.90 | 0.42 |
| **Comfort** |  |  |  | |  | |  |  |
| Follow-up | 2.07 | 0.76 to 3.38 | 0.002 | | 2.19 | | 0.89 to 3.49 | 0.001 |
| **Somatic awareness** |  |  |  | |  | |  |  |
| Follow-up | 1.09 | -0.48 to 2.67 | 0.17 | | 1.24 | | -0.35 to 2.82 | 0.13 |
