# Supplementary material for: The Effects of a Mindfulness Program on Mental Health in Students at an Undergraduate Program for Teacher Education: A Randomized Controlled Trial in Real-Life
Source: Front Psychol. 2021 Dec 6;12:722771. doi: 10.3389/fpsyg.2021.722771 (PMC8687132; doi:10.3389/fpsyg.2021.722771)
Supplement: Supplementary file 5 [file Table_5.DOCX]

**Supplemental table 5.** Associations of the mediator outcomes with the mental health outcomes. The *b* paths coefficients jf Figure 1.

| **Outcome**  **Mediator** | **PSS**  Coefficient, 95%CI, p | **SCL-5**  Coefficient, 95%CI, p | **WHO-5**  Coefficient, 95%CI, p | **BRS**  Coefficient, 95%CI, p |
| --- | --- | --- | --- | --- |
| **FFMQ** |  |  |  |  |
| **Observing** | -0.55 (-1.18 to 0.07), 0.08 | -0.02 (-0.09 to 0.05), 0.58 | -0.01 (-2.00 to 1.97), 0.99 | 0.08 (0.00 to 0.16), 0.04 |
| **Describing** | -0.26 (-0.73 to 0.21), 0.27 | -0.03 (-0.08 to 0.02), 0.27 | 1.39 (-0.04 to 2.82), 0.06 | 0.09 (0.04 to 0.15), <0.01 |
| **Acting with awareness** | -0.48 (-1.13 to 0.16), 0.14 | -0.06 (-0.13 to 0.01), 0.08 | 1.06 (-0.92 to 3.03), 0.30 | 0.01 (-0.07 to 0.09), 0.82 |
| **Non-judging of inner experience** | -0.88 (-1.35 to -0.42), <0.01 | -0.08 (-0.13 to -0.03), <0.01 | 2.71 (1.35 to 4.06), <0.01 | 0.07 (0.02 to 0.13), 0.01 |
| **Non-reactivity to inner experience** | -1.48 (-2.07 to -0.89), <0.01 | -0.17 (-0.24 to -0.10), <0.01 | 2.89 (0.87 to 4.91), 0.01 | 0.15 (0.07 to 0.24), <0.01 |
| **Total score** | -0.38 (-0.57 to -0.19), <0.01 | -0.04 (-0.06 to -0.02), <0.01 | 1.03 (0.48 to 1.59), <0.01 | 0.05 (0.02 to 0.07), <0.01 |
| **ARSQ** |  |  |  |  |
| **Discontinuity of mind** | Table 4 in paper | Table 4 in paper | Table 4 in paper | -0.06 (-0.12 to 0.00), 0.07 |
| **Theory of mind** | -0.02 (-0.55 to 0.519, 0.94 | 0.00 (-0.06 to 0.06), 0.91 | -0.25 (-1.92 to 1.43), 0.77 | -0.00 (-0.08 to 0.06), 0.89 |
| **Self** | Table 4 in paper | Table 4 in paper | Table 4 in paper | -0.03 (-0.09 to 0.03), 0.37 |
| **Planning** | 0.22 (-0.24 to 0.69), 0.34 | 0.01 (-0.04 to 0.07), 0.58 | -0.39 (-1.86 to 1.08), 0.60 | -0.01 (-0.07 to 0.05), 0.73 |
| **Sleepiness** | 0.18 (-0.35 to 0.71), 0.51 | 0.01 (-0.05 to 0.07), 0.74 | -2.05 (-3.65 to -0.45), 0.01 | 0.02 (-0.05 to 0.09), 0.54 |
| **Comfort** | Table 4 in paper | Table 4 in paper | Table 4 in paper | 0.11 (0.05 to 0.18), <0.01 |
| **Somatic awareness** | -0.47 (-1.05 to 0.12), 0.12 | -0.01 (-0.08 to 0.06), 0.75 | 1.08 (-0.77 to 2.93), 0.25 | 0.09 (0.02 to 0.16), 0.01 |

PSS: Perceived Stress Scale, SCL-5: Symptom Checklist 5, WHO-5: WHO, Well-being scale, BRS: Brief Resilience Scale, FFMQ: Five Facet Mindfulness Questionnaire, ARSQ: Amsterdam Resting State Questionnaire, CI: Confidence Interval
